# Brain Sensitivity to Exclusion is Associated with Core Network Closure

**DOI:** 10.1038/s41598-018-33624-3

**Published:** 2018-10-30

**Authors:** Joseph B. Bayer, Matthew Brook O’Donnell, Christopher N. Cascio, Emily B. Falk

**Affiliations:** 1 School of Communication, The Ohio State University, Columbus, OH USA; 2 Annenberg School for Communication, University of Pennsylvania, Philadelphia, PA USA; 3 School of Journalism and Mass Communication, University of Wisconsin, Madison, WI USA

## Abstract

Humans are driven to pursue and preserve social relationships, and these motivations are reinforced through biological systems. In particular, individual differences in the tuning of biological systems that respond to social threats may motivate individuals to seek out differently structured social environments. Drawing on a sample of adolescent males who underwent fMRI brain imaging (n = 74) and contributed Facebook data, we examined whether biological responses to a common scenario – being excluded from an activity with peers – was associated with their social network structure. We find that neural responses during social exclusion in a priori hypothesized “social pain” regions of the brain (dACC, AI, subACC) are associated with the density and transitivity of core friendship networks. These findings suggest that neural reactivity to exclusion may be one factor that underlies network “safety”. More broadly, the study shows the potential of linking social cognitive tendencies to social structural properties.

## Introduction

What psychological factors determine the shape of personal social networks? Over the past two decades, researchers have identified a number of personality traits that predict network structure in organizational and social life^1–6^. Building on this foundation, neuroimaging has the capacity to reveal additional factors that underpin social networks by examining individual differences in the tuning of brain systems^7^. In particular, given that social connectedness is reinforced through biological pathways^8^, neural sensitivity to social exclusion represents an established orientation that may be relevant to personal network structure (and vice versa). Over time, those whose brains are especially sensitive to being excluded may come to occupy the “safety” of compact, close-knit relational structures with clear expectations – i.e., network closure. In parallel, taking part in a close-knit community may also increase the costs and salience of potential exclusion. Drawing on implicit neurocognitive reactions collected via fMRI, we examine whether individual differences in neural responses during social exclusion are associated with egocentric network size (i.e., number of friends) and network closure (i.e., extent to which friends within the network are interconnected).

## Neural Responses to Social Exclusion

**Figure 1:**
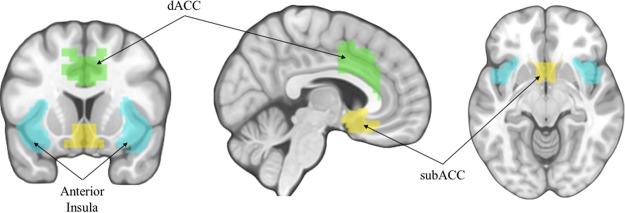
Neural regions of interest (AI, dACC, and subACC).

Experimental research has reliably shown that social exclusion damages mood, self-esteem, and sense of belonging^9^. Additionally, neuroscience research has shown that there are consistent brain regions that respond to social disconnection^10^, including the anterior insula (AI), dorsal anterior cingulate cortex (dACC)^11^, and subgenual anterior cingulate cortex (subACC) in adolescents^12^. These brain regions can also support complementary functions relevant to navigating social interactions, such as monitoring for conflict, detecting salient events and remaining alert^13–15^. Importantly, exclusion is particularly significant during adolescence, when peer relationships, and rejection from those peers, become increasingly salient and potent^16^. Extant research suggests there are links between social cognitive tendencies, brain structure and function, and social resources^17–21^, but does not account for responses to specific social contexts, such as exclusion. As such, this study focuses on adolescents’ responses within a priori hypothesized neural regions that reliably increase during exclusion relative to inclusion, as a physiological measure of the brain’s sensitivity to social exclusion. See Fig. 1.

## Full and Core Friendship Networks on Facebook

Social network theories are now commonly used to explain outcomes across disciplines^22–24^, and are increasingly relevant to social cognition and neuroscience research^17,25–27^. Kornienko *et al*. (2013) state, “Social network analysis provides powerful tools for measuring and quantifying an individual’s social ecology by focusing on his or her position in a network” (p. 386)^28^. Egocentric network (or “ego-network”) analysis thus represents a fundamental way of measuring the context surrounding an individual (or “ego”), and such socioecological perspectives are increasingly providing new insights into psychology and cognition^25,29^.

In classic social network studies, researchers often utilized name-generator methods in which participants self-report contacts^30,31^. With the advent of computer-mediated interaction, a prominent subtype of social network is a communication or interaction network^32,33^. We report on this type of network, using objectively recorded measures from Facebook’s Application Programming Interface (API), rather than self-reported ties. Facebook, in particular, occupies a central position in adolescent interaction and the site represents a large resource for social support and social capital^34–36^. We concentrate on Facebook networks because they tend to present a similar layered structure as offline networks^37,38^.

Often, network researchers discriminate between core networks and more peripheral networks^39^. Full networks on Facebook encompass all individuals with whom the ego is directly connected on the platform^40,41^, while core networks contain more proven sources of social support. These latter confidants, typically the top 5 to 15 friends of the ego, come with higher social expectations – but also provide security and trust^37,42^. Human networks are made up of an average of five “supportive” ties and ten “sympathetic” ties, each layer offering unique “tradeoffs”^37^. For instance, whereas the inner layers may provide emotional support in times of need, the outer layers may offer access to novel resources. Our study examines characteristics of both full and core networks in relation to individual differences in brain function.

## Network Size and Closure

In a Facebook friendship network, network size (or “ego-degree”) is equal to the number of accepted “friends” of the participant. By contrast, interactive links between each ego and alter afford a more dynamic measure of social relationships. In these cases, the alters who do not meet a specific criterion (e.g., amount or type of communication) are removed^33^. Here we utilize both types in the form of “full” friendship networks and “core” interaction networks. Through the complete set of friendships connected to an ego, we acquire an expansive measure of the individual’s broad social environment. Alternatively, our core networks provide a discrete compass of the individual’s primary environment based on communication logs. For both full and core networks, we concentrate on the role of closure, or the extent to which individuals within the network are interconnected. More precisely, we test two established metrics that signal overall network closure: density and transitivity^41,43^.

## Social Exclusion and Network Structure

Past research suggests neural reactivity to social exclusion is related to individuals’ social environments and social support. For example, people who interact with supportive others more often show less neural sensitivity to social exclusion^44^. Prior research has also observed a negative relationship between social pain responses in the brain and the amount of time spent with friends^45^. Similarly, people with higher sensitivity to rejection tend to make fewer friends during their freshman year of college^46^. Following this logic, increased neural reactivity within social pain regions should be associated with having fewer social resources, and thus smaller network size. In the current study, we build on past work to determine whether neural responses to exclusion are also related to objective network size on Facebook.

**Figure 2:**
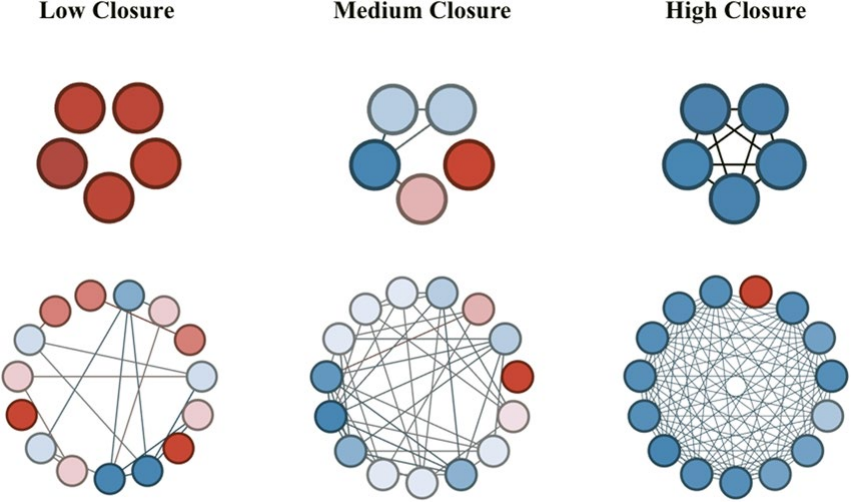
Example core networks of three participants with low, medium, and high network closure, as indexed by network density and transitivity. Two types of core networks were computed pertaining to the the five most frequent (top level) and fifteen most frequent (bottom level) interaction partners over the previous year. The network graphs are colored to differentiate friends with fewer mutual ties (red) from those with more mutual ties (blue).

The literature is less decided on how exclusion sensitivity, and neural systems in particular, may also underlie other network features such as closure. Network closure is associated with a higher concentration of close ties and connections between others in the network. Independent of differences in size, networks with greater closure are inherently more close-knit^47,48^. The friends of the ego in a more closed network are more likely to know each other and interact with one another routinely. See Fig. 2. Closure should contribute to stronger norms and enhanced trust^49,50^, codifying and clarifying the expectations of group membership. For these reasons, an interconnected network represents a certain space with clear signage, thus rendering network “safety”^50^ – at least when expectations are respected.

Following exclusion, individuals feel threatened and socially insecure^51^. In response, excluded individuals may seek out the “safety” of private areas^52^, or reach out to others – but only if acceptance seems secure^53–55^. Over time, people who are especially sensitive to social threat may “withdraw” into close-knit groups as a form of protection^46^, though this process is likely to be bidirectional (i.e., people in close-knit groups may also develop greater sensitivity to social exclusion)^56,57^. For instance, individuals may default to known cliques, in which social expectations are clearer and social investments are greater, to minimize the risk of future rejection. In practice, so long as individuals maintain good standing, they are less likely to be excluded from trusted coteries. In sum, exclusion sensitivity may be associated with retracting into social environments that are perceived as *safer*, such as close-knit circles exhibiting closure. Here we consider whether individual differences in two types of exclusion sensitivity – neural reactivity and self-reported threat– are related to closure in Facebook networks.

## Results

Our analyses examined the relationship between neural and self-reported responses to social exclusion and (1) full network size and closure, and (2) core network closure in a sample of adolescent males. The summary statistics for the primary study variables are presented in Table 1, and the bivariate correlations between them are displayed in Table 2. Full network size is equal to the total count of Facebook friends. We set the size of core networks to be the same across all participants (i.e., top 5 and top 15 friends). We then considered two forms of closure within each network: density and transitivity. Network density is equal to the proportion of actual friend connections out of all possible links among people included in the ego network. Network transitivity is equal to the proportion of completed triangles out of all possible triads, or cases in which Friend A knows Friend B and Friend B knows Friend C.

**Table 1:** Summary Statistics for Key Study Variables.

	Mean	SD	Confidence Interval	
			*Lower*	*Upper*
1. Network Size	511.85	307.63	440.58	583.12
2. Full Density	0.24	0.10	0.22	0.27
3. Full Transitivity	0.58	0.08	0.56	0.60
4. Top-15 Density	0.44	0.20	0.40	0.49
5. Top-15 Transitivity	0.68	0.16	0.65	0.72
6. Top-5 Density	0.53	0.27	0.47	0.59
7. Top-5 Transitivity	0.56	0.40	0.47	0.66
8. NTS Self-Report	3.74	1.04	3.50	3.98
9. AI_(exclusion> inclusion)_	0.00	0.61	−0.14	0.15
10. dACC_(exclusion>inclusion)_	−0.11	0.63	−0.26	0.03
11. subACC_(exclusion>inclusion)_	0.28	0.68	0.12	0.43
12. Social Pain ROI (AI + dACC + subACC)	−0.01	0.58	−0.14	0.13

**Table 2:** Bivariate Correlations of Key Study Variables.

	1	2	3	4	5	6	7	8	9	10	11	12
1. Network Size	—											
2. Full Density	−0.39**	—										
3. Full Transitivity	−0.47**	0.77**	—									
4. Top-15 Density	−0.04	0.32**	0.07	—								
5. Top-15 Transitivity	−0.02	0.21^#^	0.10	0.77**	—							
6. Top-5 Density	−0.08	0.31**	0.22^#^	0.75**	0.56**	—						
7. Top-5 Transitivity	−0.20^#^	0.25*	0.18	0.61**	0.61**	0.74**	—					
8. NTS Self-Report	−0.10	−0.13	−0.19^#^	0.09	0.04	−0.02	0.10	—				
9. AI_(exclusion>inclusion)_	0.05	0.02	0.05	0.27*	0.29*	0.22^#^	0.26*	−0.09	—			
10. dACC_(exclusion>inclusion)_	0.06	0.08	0.11	0.33**	0.35**	0.20^#^	0.25*	−0.08	0.84**	—		
11. subACC_(exclusion>inclusion)_	0.18	0.01	0.01	0.23^#^	0.28*	0.28*	0.36**	−0.11	0.64**	0.50**	—	
12. Social Pain ROI (AI + dACC + subACC)	0.09	0.05	0.08	0.32**	0.35**	0.24*	0.30*	−.0.09	0.96**	0.94**	0.70**	—

^*#*^*p* < 0.10; *p < 0.05; **p < 0.01.

In our data, two friends are connected if they are friends with each other on Facebook independent of the participant (“ego”). Thus, in a hypothetical network of five friends, the ego would have a maximally dense network if all five friends know each other, but a minimally dense network if none of them are friends on Facebook. Similarly, transitivity focuses solely on *triads* in their friends’ network: sets of three friends in which at least one knows the other two. Therefore, a fully transitive network would mean that each triad is a triangle; that is, among possible triads, the three friends always know one another. Since the ego was automatically connected to all other nodes in the network (by virtue of being friends), networks including the ego have the potential to exaggerate structural measures of interconnectedness. For this reason, density and transitivity were calculated with ego (and its edges) removed from the network.

### Correlates of Full Network Structure

The full network measures were computed from the complete friend list collected via the Facebook API. Ordinary least squares regression was used to test all of our models. In the size models, full network size was entered as the outcome variable. In the closure models, full network density and transitivity were evaluated as separate outcome variables.

#### Full Network Size

We first examined whether there was a relationship between brain responses to exclusion > inclusion and full network size, controlling for whether the participant came from sample wave one or two (see methods), as well as overall number of Facebook interactions of the individual. Neural sensitivity to exclusion was not significantly related to full network size, [β = 0.10, t(70) = 0.89, *p* > 0.37]. We next tested whether self-reported distress, i.e., reduced need satisfaction, was associated with full network size, once again controlling for sample wave and the number of Facebook interactions. Self-reported distress was also unrelated to network size, [β = −0.11, t(70) = −0.97, *p* > 0.33]. Finally, a combined model with both neural and self-report predictors entered simultaneously confirmed that neither self-reported need satisfaction nor neural responses were associated with full network size on Facebook. See Table 3 for complete results of the combined model.

**Table 3:** Exclusion Responses Associated with Full Network Size, Density, and Transitivity.

	Full Network Size		Full Network Density		Full Network Transitivity	
	β	*t* Value	β	*t* Value	β	*t* Value
**Covariates**						
Participant Wave	−0.15	−1.32	−0.09	−0.80	−0.11	−1.01
Full Network Size	—	—	−0.32**	**−2.78**	−0.48***	−4.27
Total Interactions	0.39**	**3.56**	−0.27*	**−2.36**	−0.09	−0.81
**Measures Relevant to Exclusion**						
Self−Report Need Satisfaction	−0.10	−0.87	**−0.21** ^#^	**−1.92**	−0.28*	**−2.58**
Brain Activity in Social Pain Network during Exclusion > Inclusion	0.09	0.79	0.04	0.37	0.08	0.81
R^2^	0.18** (df = 69)		0.26*** (df = 68)		0.31*** (df = 68)	

Estimates are standardized regression coefficients.

^*#*^*p* < 0.10; *p < 0.05; **p < 0.01; ***p < 0.001.

#### Full Network Closure

We next specified separate models to test the relationship between brain responses to exclusion > inclusion and (a) full network density and (b) full network transitivity, controlling for whether the participant came from sample wave one or, number of Facebook interactions, and full network size. Neural responses to exclusion were not significantly related to full network density, [β = 0.06, t(69) = 0.56, *p* > 0.57], or transitivity, [β = 0.11, t(69) = 1.04, *p* > 0.30], in their respective models. In turn, we then specified models to examine whether self-reported distress was associated with full network closure, once again controlling for sample wave and the number of Facebook interactions. Self-reported distress was marginally correlated with full network density, [β = −0.22, t(69) = −1.98, *p* < 0.052], and positively related to transitivity, [β = −0.28, t(69) = −2.68, *p* < 0.01]. Finally, we confirmed that the same pattern of results held in a model that included both neural and self-reported responses to exclusion (see Table 3 for full results of the combined model).

Last, we explored the possibility that network size might moderate the relationship between responses to exclusion and full network closure. Network size, neural responses to exclusion, and self-reported distress variables were centered and scaled for each of the models containing interaction terms. Full network size moderated the relationship between neural responses to exclusion and full network density, [β = −0.17, t(67) = −2.60, *p* < 0.02], but not transitivity, [β = −0.09, t(67) = −1.34, *p* > 0.18]. Network size also moderated the relationship between self-reported distress and full network transitivity, [β = 0.17, t(67) = 3.02, *p* < 0.004], but not density, [β = 0.11, t(67) = 1.76, *p* > 0.08]. For both neural and self-report measures, simple slopes analysis (+/−1 SD) revealed that greater reactivity to social exclusion was associated with increased closure among participants with smaller networks (i.e., neural with density, B = 0.06, t(67) = 2.49, *p* < 0.02; self-report with transitivity, B = −0.05, t(67) = −4.03, *p* < 0.001); however, among those with larger networks, increased reactivity to social exclusion was not significantly related to closure (neural with density, B = −0.02, t(67) = −1.44, *p* > 0.15; self-report with transitivity, B = 0.02, t(67) = 1.09, *p* > 0.28). All coefficients reported from the simple slopes analyses correspond to unstandardized coefficients.

### Correlates of Core Network Structure

We utilized the Facebook wall data to create more refined networks with participants’ top friends over the prior year (i.e., “core networks”). This allowed us to identify the most important friends independently from how much the participant used Facebook, as well as to directly investigate the attributes of participants’ core networks. In line with past research on core networks^37,39^, we focused on the top five and top fifteen friends, as defined in this case by the number of unique interactions on participants’ walls. In doing so, this allowed us to test how different structural features, including density and transitivity, operated at more central network layers while holding network size constant. Similar to past examinations of communication networks^58^, and Facebook in particular^38^, we observed that the top friends represented a large share of the overall interactions for each participant. Once again, network density and transitivity were evaluated in separate models as DVs.

#### Top-15 Closure

We first assessed whether there was a relationship between brain responses to exclusion > inclusion and closure among the Top-15 friends of the ego, controlling for sample wave, total interactions, full network size, and Top-15 interactions. We found that increased neural reactivity to exclusion (vs. inclusion) was associated with greater core network density, [β = 0.32, t(68) = 2.86, *p* < 0.006], as well as transitivity, [β = 0.36, t(68) = 3.22, *p* < 0.002], among the top 15 friends. Next, we specified models to examine whether self-reported distress following exclusion was associated with density among the top 15 friends, once again controlling for sample wave, total interactions, full network size, and Top-15 interactions. Self-reported distress was not significantly related to either core network density, [β = 0.03, t(68) = 0.25, *p* > 0.80], or transitivity, [β = −0.02, t(68) = −0.19, *p* > 0.85]. Finally, we confirmed that the results were parallel in combined models that included both the neural and self-reported responses to exclusion. These models showed that neural responses to exclusion – but not self-reported distress – was positively associated with Top-15 network density and transitivity. See Tables 4 and 5 for complete information on the combined models.

**Table 4:** Exclusion Responses Associated with Core Network Density.

	Core Network *Top 15 Friends*		Core Network *Top 5 Friends*	
	β	*t* Value	β	*t* Value
**Covariates**				
Participant Wave	−0.11	−0.95	−**0.20**^#^	−**1.69**
Full Network Size	0.03	0.23	0.04	0.26
Total Interactions	−0.01	−0.07	0.11	0.92
Core Network Interactions	0.16	1.11	0.39**	**2.89**
**Measures Relevant to Exclusion**				
Self−Report Need Satisfaction	0.07	0.55	−0.10	−0.83
Brain Activity in Social Pain Network during Exclusion > Inclusion	0.33**	**2.88**	0.28*	**2.50**
**R** ^**2**^	0.15^#^ (df = 67)		0.20* (df = 67)	

Estimates are standardized regression coefficients.

^*#*^*p* < 0.10; *p < 0.05; **p < 0.01; ***p < 0.001.

**Table 5:** Exclusion Responses Associated with Core Network Transitivity.

	Core Network *Top 15 Friends*		Core Network *Top 5 Friends*	
	β	*t* Value	β	*t* Value
**Covariates**				
Participant Wave	−0.19	−1.63	−0.16	−1.43
Full Network Size	−0.05	−0.38	−0.10	−0.76
Total Interactions	0.11	0.89	0.04	0.37
Core Network Interactions	0.09	0.62	0.29*	**2.21**
**Measures Relevant to Exclusion**				
Self−Report Need Satisfaction	0.01	0.11	0.03	0.29
Brain Activity in Social Pain Network during Exclusion > Inclusion	0.36**	**3.20**	0.34**	**3.13**
**R** ^2^	0.17* (df = 67)		0.23** (df = 67)	

Estimates are standardized regression coefficients.

^*#*^*p* < 0.10; *p < 0.05; **p < 0.01; ***p < 0.001.

#### Top-5 Closure

We also examined the relationship between brain responses to exclusion > inclusion and closure among the top 5 friends of the ego, controlling for sample wave, total Facebook interactions, full network size, and Top-5 interactions. Similar to the Top-15 models, we found that neural reactivity was related to increased network density, [β = 0.29, t(68) = 2.59, *p* < 0.02], and transitivity, [β = 0.34, t(68) = 3.14, *p* < 0.003], among the top 5 friends in separate models. Also paralleling the Top-15 models, self-reported distress was unrelated to core network density, [β = −0.12, t(68) = −1.02, *p* > 0.31], and transitivity, [β = 0.00, t(68) = 0.00, *p* > 0.99], with sample wave, total interactions, full network size, and Top-5 interactions entered as covariates. Likewise, as displayed in Tables 4 and 5, we observed parallel relationships in our combined models that included both neural responses and self-reported distress.

#### Robustness check

Due to moderate non-normality in our measures of core network closure, we conducted rank regression versions of the combined models (see Supplementary Materials). These added models offered convergent evidence with the OLS models.

#### Whole brain searches

We also examined whether regions outside of our a priori hypothesized regions of interest were associated with our key social network variables. Consistent with our a priori hypotheses, activity in dACC was associated with the density and transitivity of the Top-15 networks (see Supplemental Materials). Additional activity was observed in parts of lateral prefrontal and parietal cortices for density and transitivity of the Top-15 networks across thresholding strategies, with more widespread activations under less conservative thresholding strategies. Activity in insula, as well as additional portions of pre-frontal cortex and temporal cortex were associated with Top-5 Transitivity. No regions survived multiple comparisons correction at the whole brain level for models linking brain activity to the density of the full networks or Top-5 networks, or transitivity of the full networks.

## Discussion

In this investigation, self-reported sensitivity to exclusion was associated with full network closure. Neural responses were associated with closure at the core network level, such that adolescents who showed stronger responses during exclusion exhibited higher closure among their top friends. In other words, greater self-reported and neural sensitivity to exclusion is related to interacting with more close-knit circles. Moreover, we also observed that responses to exclusion were associated with full network closure – but only for those with smaller Facebook networks. Combined, the results strengthen the notion that individuals with elevated exclusion sensitivity may gravitate to more close-knit, and thus “safer”, social environments. By surrounding oneself with a tightly woven set of friends with known expectations, one may minimize the risk of being isolated during daily life. Concurrently, being embedded in close-knit network structures may heighten sensitivity to signals of being excluded, since the costs of exclusion may multiply when friends know one another.

Our results linking self-reported distress following exclusion to full network closure add to a growing body of work tying exclusion to social network features. For example, freshman students who were higher in rejection sensitivity ended the school year with a less diverse set of friends^46^. Nonetheless, a variety of different mechanisms may contribute to these links, such as in-group favoritism or exaggerated preference for homogeneity. In turn, future work should strive to measure network preferences in combination with sensitivity to exclusion. Our findings also expand upon recent fMRI research, which has started to examine the links between neurocognition and social network characteristics, including size and other network dimensions that are germane to closure (e.g., brokerage, diversity). For example, the diversity of a person’s social roles in a social network is positively correlated with white matter integrity^20^. Also, more popular people are more sensitive to others’ social network popularity (measured within the brain’s valuation system including vmPFC, ventral striatum, and amygdala) – and better at detecting others’ actual popularity^27^. Hence, our study affirms the relevance of social cognitive differences for network structure beyond size, along with the potential for network dimensions to moderate core cognitive processes^25,26^.

The conditional relationship between exclusion sensitivity, network size, and network closure complements prior work suggesting that people may hold several socioemotional motives for network engagement at the same time. For example, two competing individual motivations – “safety” vs. “efficacy” – are theorized to influence personal network structure^50,59^. Accordingly, individuals are motivated to both (1) reinforce their most trusted circles and (2) seek out new leverage positions in an entrepreneurial fashion. Whereas efficacy is tied to network brokerage and decreased closure, the safety motivation is tied to increased closure, including greater density and transitivity^50^. In turn, individuals are driven to pursue both network structures – but toward different ends. In the current case, we show that those who show the greatest self-report and neural responses to exclusion may tend to retreat to more closed groups with well-defined norms, and this proclivity may shape their social networks over time. We thereby bolster the idea that reactivity to social threat may underlie ego-networks tuned toward “safety”, particularly for those with smaller overall networks.

Within the brain, we also observed relationships with core network structure. Past research demonstrates that dACC^11^, AI^60^, and subACC^12^ reliably increase during social exclusion compared to inclusion. Our results suggest that individual differences in sensitivity within these regions are also associated with core social network structures that people inhabit. Results from our whole brain search suggest particularly robust involvement of the dACC. Indeed, among the three a priori defined ROIs, the dACC exhibited the strongest relationship to core network closure in three out of four models. From a cognitive perspective, the core regions of interest associated with the effects we observe, and dACC in particular, is implicated in conflict monitoring^61^, as well as other mental processes such as salience detection^15^, tonic alertness^14^, task-set maintenance^62^, anxiety, and distress that together suggest an alarm-like function^13,63^. In other words, given that social exclusion is costly in an evolutionary context^10^, as well as a modern context (e.g., “fear of missing out”)^64^, brain activity that detects potential conflicts, monitors for salient threats, and more generally responds to social distress, is thought to keep individuals motivated to stay connected to their groups. Our data highlight that individual differences in brain responses within this system may shape and be shaped by the types of social networks people occupy. For those who respond more strongly to negative cues from others, it may be safer to uphold membership in a close-knit group (vs. discrete friends).

By contrast, we found no evidence that the number of friends in an online social network, i.e., objectively-logged larger networks, is associated with either self-reported or neural responses to social exclusion. It is possible that reactivity to exclusion is associated with different preferences for the quality, rather than quantity, of relationships. In particular, previous research focusing on offline support and raw time spent with friends demonstrates the power of social activity to buffer reactivity to exclusion^44,45^. Although these findings appear contradictory on the surface, objectively logged measures of Facebook network size likely tap into different qualities than time spent with friends offline. In addition, the study of time spent with friends examined friendships two years prior as a predictor of neural reactivity to exclusion, whereas our study examined aggregate network structure over a yearlong period. It is also worth noting that different social context factors (e.g., time spent with different friends vs. group membership with strong norms) may be associated with different neural responses to exclusion. As such, future research should aim to triangulate measures of social network characteristics, including deeper investigations into temporal dynamics, subjective vs. objective assessment of network properties, and the quality vs. quantity of relationships. Triangulation may also help reconcile our findings with research that observed links between self-reported network size and neuroimaging measures, such as studies linking grey matter volume of social cognitive regions and greater functional connectivity between the amygdala and cortical regions associated with social perception and affiliation^17,65^. Finally, as noted above, we observed that network size moderates the relationship between both self-report and neural responses to exclusion and network closure. As such, our findings reaffirm the importance of considering the structure and function of social networks in combination with size.

In parallel, our results highlight the potential to identify discrete roles of core networks and full networks. For our sample of adolescents, the networks made up of top friends (vs. total friends) were more strongly related to neural responses during exclusion (vs. inclusion). This is consistent with other research showing that the number of “actual friends” on Facebook, as opposed to total friends, can be predictive of social outcomes^66^. By contrast, the closure of full networks was most strongly associated with self-reported distress following the exclusion episode. Post-hoc analyses offered some insight about where the processes may overlap. Specifically, we found evidence that network size may moderate the relationship between exclusion reactivity measured both during exclusion with fMRI and after exclusion with self-report, and full network closure. For individuals with smaller full Facebook networks, the relationship between both measures of reactivity to exclusion and full network closure matched that of the relationship between neural reactivity and core network closure. This qualified relationship between exclusion sensitivity and network closure indicates the need for more nuanced perspectives, particularly when explaining the structure of peripheral layers. The finding is also an important reminder that large-scale online networks reflect numerous and heterogeneous factors, ranging from the number of places a person has lived to their motivations for friending or following others. In line with prior theoretical approaches^67^, we also argue that personality inclinations can have separate effects on the structural features across different types of personal networks. By testing full and core networks side-by-side, we show that outer and inner circles can have distinct correlates with key individual differences.

More broadly, our findings strengthen calls for network measures to be incorporated into studies of psychological and cognitive science^7,49,68^, as well as for greater links between sociological and cognitive neuroscience perspectives^69,70^. Indeed, our data add another layer of nuance to prior network research evaluating the “social brain” hypothesis, or the idea that brain processing has evolved to keep track of complicated social worlds^18,21^. Thus far, the majority of research linking social network measures and psychological tendencies has measured individual characteristics with self-report methods^3,68^. The separate relationships observed for the two measures of responses to exclusion (fMRI and self-report NTS scale) affirms the usefulness of combining self-report and neural methods in the study of social interaction, as fMRI can capture experiences that may complement self-report, and visa-versa^71^. Specifically, the implicit neural measures were reliably related to core network closure, while the self-reported distress was associated more robustly with full network closure. Despite the two measures providing generally parallel implications – reactivity to social exclusion being associated with network closure – future work should attempt to clarify whether discrete mechanisms exist.

Of course, the limitations of the current dataset and correlational analyses provide important avenues for future research to consider. For example, we focused on adolescent, male Facebook users who volunteered information about their profiles. Although most participants chose to contribute their digital trace data, it is possible that this self-selection process biases our sample. It is also possible that the individual differences in network features are indexing a hidden variable unintentionally (e.g., user motivations, life changes, geographical shifts, etc.), or that different results would be observed using other samples^72^. Additionally, it is possible that our logged collection of interactions unintentionally captures some individuals who are more or less meaningful to the ego’s core friendship network (e.g., family members). For these reasons, future research should evaluate whether other types of networks (e.g., Face-to-Face, Calling, Twitter) relate to *in vivo* cognitive tendencies, and whether our results here maintain over time and generalize to other populations. Last, the results should be interpreted with the usual caution regarding reverse inference in neuroimaging research^73^, given that multiple functions underlie our brain regions of interest and hence the psychological interpretation of the brain reactivity is open to different interpretations^74–76^.

Our theoretical framework linking exclusion sensitivity to network structure reflects past research on social networks, which have generally treated personality factors as *predictors* of network structure^3^. Nonetheless, in addition to individuals shaping their social environments, social environments also affect individuals, and our data cannot untangle the directionality of these complex socio-psychological processes. In other words, there are likely bidirectional relationships between life experiences, network properties, and brain responses. For example, being part of a dense community may translate into greater sanctions for negative behavior from the group^77,78^. Those who are part of such networks may be more vigilant to potential rejection since the consequences may be greater. Future research is thus needed to establish causality – the extent to which more reactive individuals embed themselves in closed networks, the extent to which interacting with a closed network sensitizes individuals, and the extent to which the two directions mutually reinforce one another. Moreover, some dense networks may actually serve to buffer individual responses to social exclusion; recent work suggests that the relationship between density and well-being depends on the level of self-affirmation^79^. A self-affirming dense network increases self-efficacy and self-esteem, whereas a disaffirming dense environment can undermine well-being. In all likelihood, these relationships with ego-network structure are reciprocal and conditional^57,80^.

In total, we found that adolescents who reacted more intensely to exclusion (via fMRI) tended to have denser core friendship networks, but did not differ in the number of friendship connections within their networks. Self-reported reactions to exclusion were also positively associated with more closed full friendship networks on Facebook – but only for those with smaller networks. Experiencing stronger reactivity to social exclusion may lead individuals to seek out the certainty of an interconnected group, rather than the uncertainty of disconnected friendships, and visa-versa. Our findings thus suggest that exclusion sensitivity is related to the structure, rather than the sum, of interaction partners. As such, this study extends our understanding of the social cognitive correlates of network structure. Further, our findings underscore the promise of combining neuroimaging with network science to connect social cognitive processes and social network properties. Just as the social network literature has linked structural features to a broad range of informational, organizational, and health outcomes, we are now poised to make stronger links to personal cognition.

## Methods

### Participants

Participants were recruited from a list of recently licensed teenage drivers provided by the Secretary of State in Michigan, U.S.A., as part of a larger study examining adolescent male driving behavior and susceptibility to peer influence^81,82^. As such, the sample was homogenous with regard to age (all participants were between 16 and 17 years old), gender (male), and race (White). Participants were collected in two groups during 2012 (n = 35; *M* = 16.9 years, *SD* = 0.47 years) and 2013 (n = 70; *M* = 16.9, *SD* = 0.30) a year later. Post-hoc analyses confirmed the two participant waves did not differ significantly on demographics or neural response to exclusion and are combined for the purpose of this investigation (see Supplemental Materials). Additionally, all regression models included a covariate for sample wave to account for any unknown differences between the waves. In addition to completing an fMRI session, a subsample of 74 participants also provided logged Facebook network data. Two participants had missing portions of their imaging data, so they were removed from all analyses. The remaining participants from the larger neuroimaging sample (n = 29) either chose not to contribute data from their Facebook profiles when asked to do so voluntarily or experienced technical problems that undermined logged data collection. Participants met standard fMRI and driving simulator inclusion criteria, such that all participants were right-handed, did not suffer from claustrophobia, were not currently taking any psychoactive medications, had normal (or corrected to normal) vision, did not have metal in their body that was contraindicated for fMRI, and did not typically experience motion sickness.

### Procedures

All study procedures were approved by the University of Michigan IRB and performed in accordance with relevant guidelines and regulations. Informed consent was obtained for all participants. Specifically, the teenage participants and their parents gave verbal and written assent/consent, respectively, before beginning the study. All data collection for the current report was completed during one appointment. All participants completed Cyberball^83^, a game in which they are socially excluded, during an fMRI session, as well as a post-scan self-report measure of distress (Need Threat Scale; NTS)^84^ in response to the exclusion task. Participants were later asked to provide access to their logged Facebook network data.

#### fMRI Session

Participants played the computerized game “Cyberball” while we monitored neural activity throughout the brain using fMRI. This manipulation has been shown to produce negative feelings associated with ostracism in many replications^85^ (see Supplemental Materials). Parallel analyses drawing on this dataset have investigated changes in neural connectivity during exclusion and inclusion in relation to full network density^86^; however, no prior reports have examined average changes in brain activation nor core network properties, or the interaction between network size and structure.

#### Self-Reported Distress Following Exclusion

The Need Threat Scale (NTS) was administered after the participants exited the fMRI scanner in order to evaluate self-perceptions of the social exclusion scenario. Responses were assessed on a 7-point scale ranging from 1 (*strongly disagree*) to 7 (*strongly agree*). Participants answered 20 questions (e.g., *I think that my participation in the game was useful, I had the idea that I had the same value as the other players*, and *I had the feeling that I belonged to the group during the game*). Higher scores on the NTS indicate greater need *satisfaction*, or less self-reported distress following the manipulation. We averaged each sub-scale in line with previous work and tested the reliability across the four dimensions, confirming that the full scale had good reliability (Cronbach’s α = 0.89).

#### Facebook Data

After completing the fMRI session, each participant was asked whether he had a Facebook account. If so, he was asked whether he would be willing to contribute data from his personal wall feed to the study using an app that automatically logs historical data from the participant’s profile. See Supplemental Materials.

#### fMRI data acquisition

Imaging data were acquired using a 3 Tesla GE Signa MRI scanner. One functional run was recorded for each participant (251 volumes). Functional images were recorded using a reverse spiral sequence (TR = 2000ms, TE = 30 ms, flip angle = 90°, 43 axial slices, FOV = 220 mm, 3 mm thick; voxel size = 3.44 × 3.44 × 3.0 mm). A set of high resolution in plane structural images was recorded (43 slices; slice thickness = 3 mm; voxel size = 0.86 × 0.86 .3.0 mm) to facilitate co-registration and normalization. In addition, a set of high-resolution structural T1-weighted anatomical SPGR images was acquired (124 slices; slice thickness = 1 mm; voxel size = 1.02 × 1.02 × 1.2 mm). Behavioral responses (i.e., Cyberball throws) were executed using a scanner compatible five-finger glove.

### Data Analysis

#### fMRI preprocessing and modeling

Functional data were pre-processed and analyzed using Statistical Parametric Mapping (SPM8, Wellcome Department of Cognitive Neurology, Institute of Neurology, London, UK) according to standard pre-processing stream (see Supplemental Materials). Data were modeled for each subject using the general linear model as implemented in SPM8. Three trial phases were modeled with one regressor each: social inclusion (89 TRs, 178 seconds), social exclusion (89 TRs, 178 seconds). These phases were each modeled as single blocks and convolved with the synthetic hemodynamic response as provided by SPM. The six rigid-body translation and rotation parameters derived from spatial realignment were also included as nuisance regressors. Data were high-pass filtered with a cutoff of 128 s.

#### Social Pain Regions of Interest

We focused on a priori hypothesized regions of the brain that have been previously associated with distress during exclusion: dACC, anterior insula, and subACC^87,88^. See Supplemental Materials for anatomical definitions. Percent signal change scores were extracted from the contrast exclusion > inclusion for the ‘social pain’ ROI as a whole for our regression models, as well as the individual ROIs (see Table 2).

#### Interaction Measures

Separate measures were computed in order to account for individual differences in Facebook use as well as to identify “Top Friends” according to the degree of Facebook activity. *Total Interactions*, which is controlled for in all models, represents the complete number of interactions (mentions, comments, likes, etc.) with all friends of each participant (*M* = 858.68, *SD* = 991.23, Min = 18, Max = 4856). By contrast, *Core Interactions* represents the proportion of total interactions occurring with the friends in participants’ Top-5 (*M* = 0.20, *SD* = 0.10) or Top-15 (*M* = 0.38, *SD* = 0.16) friend networks. In core network models, we thus included covariates for both complete and core network interaction levels.

#### Network Measures

The logged Facebook data, including data about participants’ Facebook friends and friends-of-friends, was used to create the ego-network measures (size, density, transitivity). Full network size reflected the number of discrete nodes connected to the ego, or n. Density is equal to the proportion of connected nodes out of total possible links, or d = 2 m/(n * n − 1), where m denotes the number of edges and n denotes the number of nodes. Transitivity is equal to the proportion of closed triangles in cases when two links share a vertex, or t = 3 * q/r, where q denotes the number of close triplets and r denotes the number of triads (two edges with a shared vertex).

#### Testing Hypotheses

Ordinary least squares (OLS) regression models were run in R to test our hypotheses. Due to moderate non-normality in some of the core network variables, we also ran each model as a rank regression to confirm the findings were robust. Network analyses for density and transitivity were conducted using NetworkX in Python. Rank regression models were run using the Rfit package in R (Supplementary Materials). Our primary full model was specified as Y = β_0_ + β_1_W + β_2_F + β_3_S + β_4_D + β_5_N + [β_i_M_i_] + ε, in which Y denotes the network structural outcome, W denotes the wave of data collection, F denotes the amount of participant interactions on Facebook, S denotes the size of participant networks, D denotes the self-reported distress following exclusion, N denotes neural responses to exclusion > inclusion, and M_i_ denotes models specific terms outlined below (i.e., Full Network Size in closure models, and Core Interactions in core network models). We also confirmed that the results for our primary predictors of interest (self-report and neural responses to exclusion) were similar when included in separate models: Y = β_0_ + β_1_W + β_2_F + β_3_S + β_4_D + [β_i_M_i_] + ε and Y = β_0_ + β_1_W + β_2_F + β_3_S + β_5_N + [β_i_M_i_] + ε. As such, all regression models controlled for Sample Wave (1 or 2) to account for potential confounds in data source and Total Interactions to account for individual differences in Facebook use. Additionally, all closure models controlled for Full Network Size and all core network models controlled for Core Interactions, as specified above.

#### Exploratory Whole Brain Analyses

We also conducted a set of exploratory whole brain models to determine if additional neural processes associated with exclusion were related to network size and closure that extended beyond our hypothesized ROI analyses. These additional analyses independently regressed each focal social network variable above onto the contrast (exclusion > inclusion) during the Cyberball task. Whole brain analyses were reported for clusters that were significant using cluster correction at p < 0.001, k = 79, together corresponding to p < 0.05, corrected for FWE based on parameters derived from 3dClustSim, using smoothness parameters (16.0, 18.0, 18.5 mm) estimated from the residuals of each statistical map (updated, July 2016). In addition, whole brain analyses were also examined using the default FDR threshold implementation in SPM8, with a threshold of pFDR < 0.05 (K > 20), corrected. This threshold combination balances concerns about type I error^89,90^ and concerns about type II error. See Supplementary Materials for additional details and results.

## Electronic supplementary material

Supplementary Materials

## Data Availability

The datasets generated and/or analyzed during the current study are available from the corresponding author on reasonable request.
