# Supplementary material for: Brain Sensitivity to Exclusion is Associated with Core Network Closure
Source: Sci Rep. 2018 Oct 30;8:16037. doi: 10.1038/s41598-018-33624-3 (PMC6207694; doi:10.1038/s41598-018-33624-3)
Supplement: Supplementary file 1 — Supplementary Materials [file 41598_2018_33624_MOESM1_ESM.docx]

**Brain Sensitivity to Exclusion is Associated with Core Network Closure**

Joseph B. Bayer ^1^
Matthew Brook O’Donnell ^2^
Christopher N. Cascio ^3^

Emily B. Falk ^2^

^1^ School of Communication, The Ohio State University, Columbus, OH, USA

^2^ Annenberg School for Communication, University of Pennsylvania, Philadelphia, PA, USA
^3^ School of Journalism and Mass Communication, University of Wisconsin, Madison, WI, USA

­­­

*Correspondence:*

Dr. Joseph B. Bayer

School of Communication

The Ohio State University

154 N. Oval Mall

Columbus, OH 43210, USA

bayer.66@osu.edu

Dr. Emily B. Falk

Annenberg School for Communication

University of Pennsylvania

3620 Walnut Street

Philadelphia, PA 19104, USA

falk@asc.upenn.edu

**Supplementary Materials**

*fMRI Processing.* To allow for the stabilization of the BOLD signal, the first four volumes (eight seconds) of each run were discarded prior to analysis. Functional images were despiked using the 3dDespike program as implemented in the AFNI toolbox. Next, data were corrected for differences in the time of slice acquisition using since interpolation; the first slice served as the reference slice. Data were then spatially realigned to the first functional image. We then co-registered the functional and structural images using a two-stage procedure. First, in-plane T1 images were registered to the mean functional image. Next, high-resolution T1 images were registered to the in-plane image. Following co-registration, the high-resolution T1 images were segmented into white and gray matter allowing the skull to be removed. Structural and functional images were then normalized to the skull-stripped MNI template provided by FSL (“MNI152_T1_1mm_brain.nii”). In the final pre-processing step the functional images were smoothed using a Gaussian kernel (8 mm FWHM).

*Anatomical ROIs.* Anatomical ROIs were constructed in Wake Forest University Pickatlas toolbox within SPM ^1^, taking definitions from the Automated Anatomical Labeling Atlas (AAL) ^2^, Brodmann areas, and manual tracing, intersected with x, y, z bounds. MarsBar ^3^ was used to convert these anatomical images to regions of interest (ROIs). The AI ROI was defined as all voxels within the left and right insula masks provided by PickAtlas that were anterior to the y = 0 plane. The dACC ROI was defined as the union of Brodmann areas 24 and 32 (dilated to 2mm), as well as the anterior, middle, and posterior cingulate masks from the AAL atlas, minus Brodmann areas 8 and 9. Finally, the ROI is restricted to the voxels bounded by (x = -16 to 16, y = 0 to 33, and z = 6 to 52). The subACC ROI was manually traced to include regions of the cingulate and paracingulate cortices ventral to the body of the corpus callosum and posterior to the genu. The social pain network was constructed from the union of the AI, dACC, and subACC ^4^.

*fMRI Session.* For the social exclusion manipulation (Cyberball), subjects who participated in the first data collection period met two age-matched confederates at the beginning of the study and were told that they would be participating in some tasks as a group later in the session. Subjects who participated in second data collection period of the study were shown a login screen in which the subject and two other participants “joined” the virtual game. During the Cyberball game, all participants were shown the visual representation of three people: their own hands and two other avatars with the names of the other players above them. In actuality, the participants all interacted with a preset computer program that mimicked the experience of playing catch with two others. The task was made up of two three-minute rounds. In the first round (inclusion), the participant was included equally in the passing from other players. In the second round (exclusion), the other players only threw to one another.

*Facebook Wall Data Collection.* There were no penalties for individuals who preferred not to provide wall data and all included participants provided informed assent, along with informed consent from their parents. In some cases, we encountered technical difficulties that precluded participants from volunteering their personal Facebook data. For individuals who volunteered Facebook data, the Facebook API was used to programmatically collect information about their network including the number of friends, the connections between their friends, and a record of their recent interactions through wall posts, comments, and likes. The names of all Facebook friends were removed during collection and stored in a secure database. All Facebook identifiers (for people, messages, groups and connections) were encrypted using a one-way MD5 hash, which makes it impossible to go back from hashed ID to the original ID.

**Table 6** *Summary Statistics for Key Study Variables by Sample Wave*

** indicates a significant difference between waves*

|  | **Wave 1 (N = 21)** | | **Wave 2 (N = 53)** | |
| --- | --- | --- | --- | --- |
|  | *Mean* | *SD* | *Mean* | *SD* |
| 1. Network Size | 557.10 | 292.62 | 493.92 | 314.27 |
| 2. Full Density | 0.25 | 0.10 | 0.24 | 0.11 |
| 3. Full Transitivity | 0.58 | 0.07 | 0.58 | 0.09 |
| 4. Top-15 Density | 0.49 | 0.21 | 0.43 | 0.20 |
| 5. Top-15 Transitivity | 0.73 | 0.12 | 0.66 | 0.16 |
| 6. Top-5 Density | 0.60 | 0.29 | 0.50 | 0.26 |
| 7. Top-5 Transitivity | 0.67 | 0.38 | 0.52 | 0.40 |
| 8. NTS Self-Report* | 4.20 | 1.11 | 3.56 | 0.96 |
| 9. AI Contrast | 0.04 | 0.83 | -0.01 | 0.51 |
| 10. dACC Contrast | -0.16 | 0.89 | -0.09 | 0.51 |
| 11. subACC Contrast | 0.50 | 0.80 | 0.19 | 0.61 |
| 12. Social Pain Contrast | 0.03 | 0.79 | -0.02 | 0.48 |

*Sample Waves.* The summary statistics for our key study variables, broken down by data collection wave (1 or 2), are displayed in Table 7 below. We also tested whether the two sub-samples differed significantly for our primary predictors (neural social pain contrast, need threat scale) and Facebook activity. A Welch Two-Sample T-Test revealed that participants in the first wave (*M* = 4.20, *SD* = 1.11) reported higher need satisfaction than those in the second wave (*M* = 3.56, *SD* = 0.96), t(32.7) = 2.34, p < 0.03. As such, we controlled for sample wave in all of our models. Conversely, Welch two-sample t-tests showed that the social pain responses in the brain did not differ significantly between wave one (*M* = 0.034, *SD* = 0.788) and wave two (*M* = -0.023, *SD* = 0.476), t(66.21) = -0.31, p > 0.75. There was also not a significant difference in the number of Facebook interactions between participants in the first wave (*M* = 728.57, *SD* = 584.23) and the second wave (*M* = 910.23, *SD* = 1112.88), t(66.21) = -0.91, p > 0.36.

*Rank Regression.* Since our measures of core network closure exhibited moderate non-normality, we replicated the combined OLS models reported in Tables 4 and 5 using rank regression through the rfit package in R. Specifically, we examined whether neural and self-reported responses to exclusion were related to core network closure, controlling for sample wave, total number of Facebook interactions, full network size, and the proportion of total interactions made up by core network engagement.

In the Top-15 networks, rank regression models showed that neural responses following exclusion maintained a positive relationship to Top-15 network density, [B = 0.12, t(67) = 3.02, p < 0.004], and transitivity, [B = 0.09, t(67) = 2.92, p < 0.005]. For both models, self-reported distress was not a significant predictor of network structure (p’s > 0.72). For the Top-5 networks, rank regression analyses also revealed significant effects for neural activity in the social pain ROI predicting density, [B = 0.14, t(67) = 2.48, p < 0.02] and transitivity [B = 0.23, t(67) = 2.65, p < 0.01]. Paralleling the OLS regressions, self-reported distress following exclusion was not a significant predictor in the Top-5 rank models (p’s > 0.46). Taken together, the rank regression models provided consistent results with our OLS models.

*Interaction Models related to* *Full Networks.* The significant interactions between full network size and responses to social exclusion predicting full network closure are displayed in Figure 4. As shown in the conditional coefficient plots, full network size moderates the relationship between responses to social exclusion and full network closure (neural with density; self-report with transitivity). Note: the plots go in opposite directions because higher numbers on the need threat scale indicate greater need satisfaction (i.e., *less* threat).


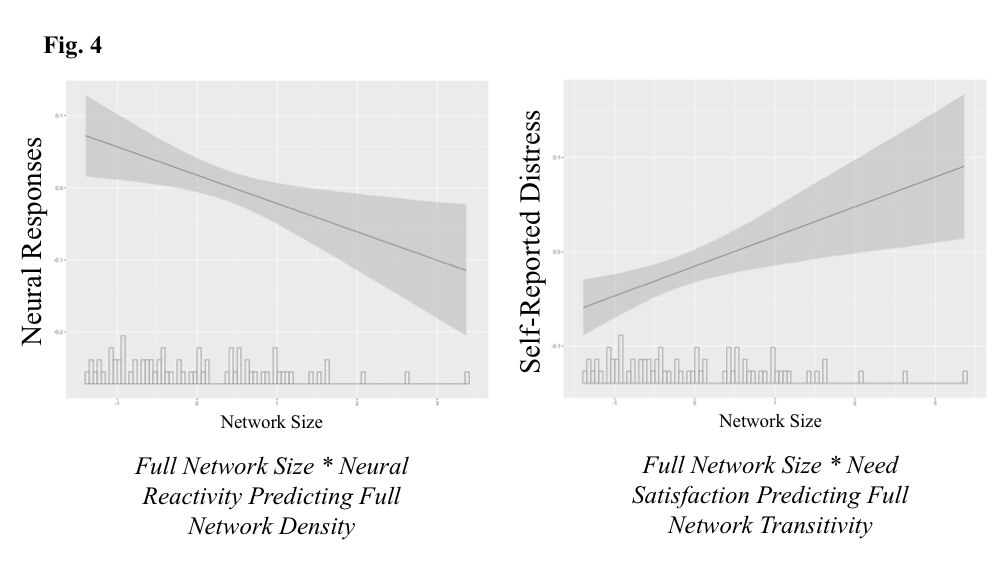


*Exploratory Whole Brain Results.* As reported in Table 7 below, whole brain analyses were computed to document regions outside of the core ROIs that were also associated with social network measures. No regions survived cluster correction for any of the full network measures using the default/ built in FDR correction using pFDR < .05, k > 20 in SPM8, nor using cluster-extent thresholding using 3dClustSim Monte Carlo simulation to achieve a whole-brain familywise error rate (FWE) of p<.05, with a primary threshold of p <.001, a cluster extent of k = 79, and smoothness parameters (16.0, 18.0, 18.5 mm) estimated from the residuals of each statistical map. Using both FDR and FWE correction (as described above), we observed activations in the Top-15 network closure metrics (transitivity, density), as well as Top-5 transitivity (but not density). Displayed below, Table 7 lists peak voxels using FWE correction according to 3dClustSim. All regions listed below also survived FDR p<.05, k>20, but with larger clusters (as this method is less conservative).

**Table 7** *Whole Brain Analyses Associated with Networks (FWE, 3DClustSim)*

| Top-15 Density (p = .001, k = 79, corresponding to p<.05, FWE) | | | | | | |
| --- | --- | --- | --- | --- | --- | --- |
|  | | | | | | |
| **Regions** | **Hemisphere** | **x** | **y** | **z** | **k** | **t(74)** |
| Precentral Gyrus/Middle  Frontal Gyrus | L | -33 | 1 | 58 | 85 | 4.05 |
| PCC/Precuneus | R/L | 5 | -50 | 37 | 141 | 4.32 |
| DMPFC/dACC | R/L | 5 | 32 | 46 | 126 | 3.79 |
| Middle Frontal Gyrus/ Anterior Insula | R | 25 | 19 | 34 | 133 | 3.91 |
| Caudate | R/L | -6 | 1 | 22 | 106 | 3.99 |
| DLPFC | R | 29 | 50 | 16 | 132 | 4.04 |
| Middle Temporal Gyrus/  Inferior Temporal Gyrus | L | -61 | -57 | -5 | 142 | 4.86 |

| Top-15 Transitivity (p = .001, k = 79, corresponding to p<.05, FWE) | | | | | | |
| --- | --- | --- | --- | --- | --- | --- |
|  | | | | | | |
| **Regions** | **Hemisphere** | **x** | **y** | **z** | **k** | **t(74)** |
| Caudate/Thalamus | R/L | -6 | 1 | 25 | 133 | 4.14 |
| dACC/DMPFC/DLPFC/SMA/  Inferior Frontal Gyrus | R/L | 29 | 8 | 25 | 2201 | 5.05 |
| Inferior Temporal Gyrus/Middle  Temporal Gyrus/Fusiform Gyrus | R | 46 | -71 | -14 | 371 | 4.99 |
| Inferior Temporal Gyrus/TPJ/PCC/ Precuneus/Angular Gyrus/Middle  Temporal Gyrus | R/L | -61 | -57 | -5 | 2791 | 5.47 |

| Top-5 Transitivity (p = .001, k = 79, corresponding to p<.05, FWE) | | | | | | |
| --- | --- | --- | --- | --- | --- | --- |
|  | | | | | | |
| **Regions** | **Hemisphere** | **x** | **y** | **z** | **k** | **t(74)** |
| Postcentral Gyrus | L | -26 | -40 | 70 | 125 | 5.82 |
| Middle Temporal Gyrus/ Medial Temporal Lobe | R | 35 | -60 | 4 | 136 | 5.57 |
| Insula/Inferior Frontal Gyrus | R | 53 | 12 | 1 | 124 | 4.09 |
| Inferior Temporal Gyrus | L | -54 | -64 | -11 | 84 | 4.23 |
| Fusiform Gyrus/Cerebellum | R | 25 | -78 | -26 | 86 | 3.83 |

3D rendering of the clusters identified in the whole brain analyses are displayed below, using the more conservative cluster correction of p < .001, k = 79, corresponding to p<.05, FWE corrected, based on 3D ClustSim. Color bar represents t-values.

**Fig. 5a** *Whole Brain Activation with Top-15 Density (FWE, 3DClustSim;* p = .001, k = 79, corresponding to p<.05, FWE)

**
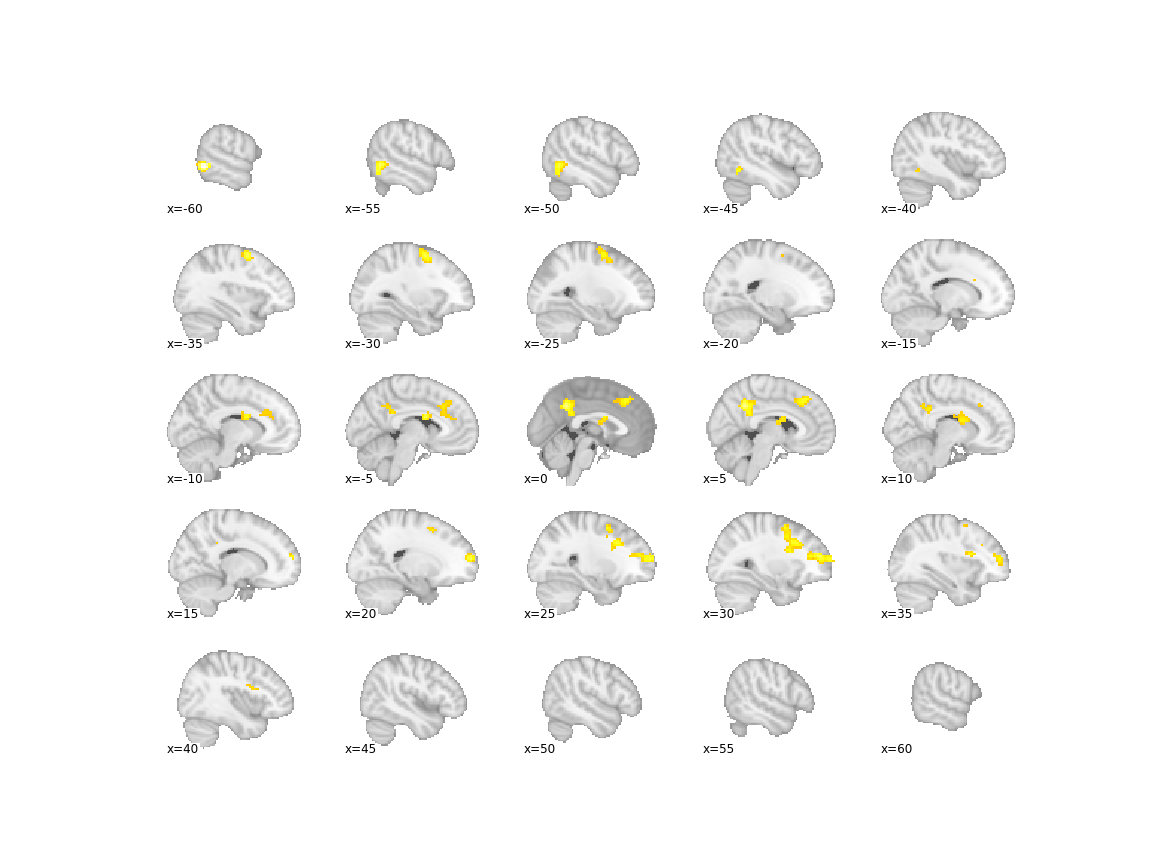
**

**
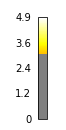
**

**Fig. 5b** *Whole Brain Activation with Top-15 Transitivity (FWE, 3DClustSim;* p = .001, k = 79, corresponding to p<.05, FWE)

**
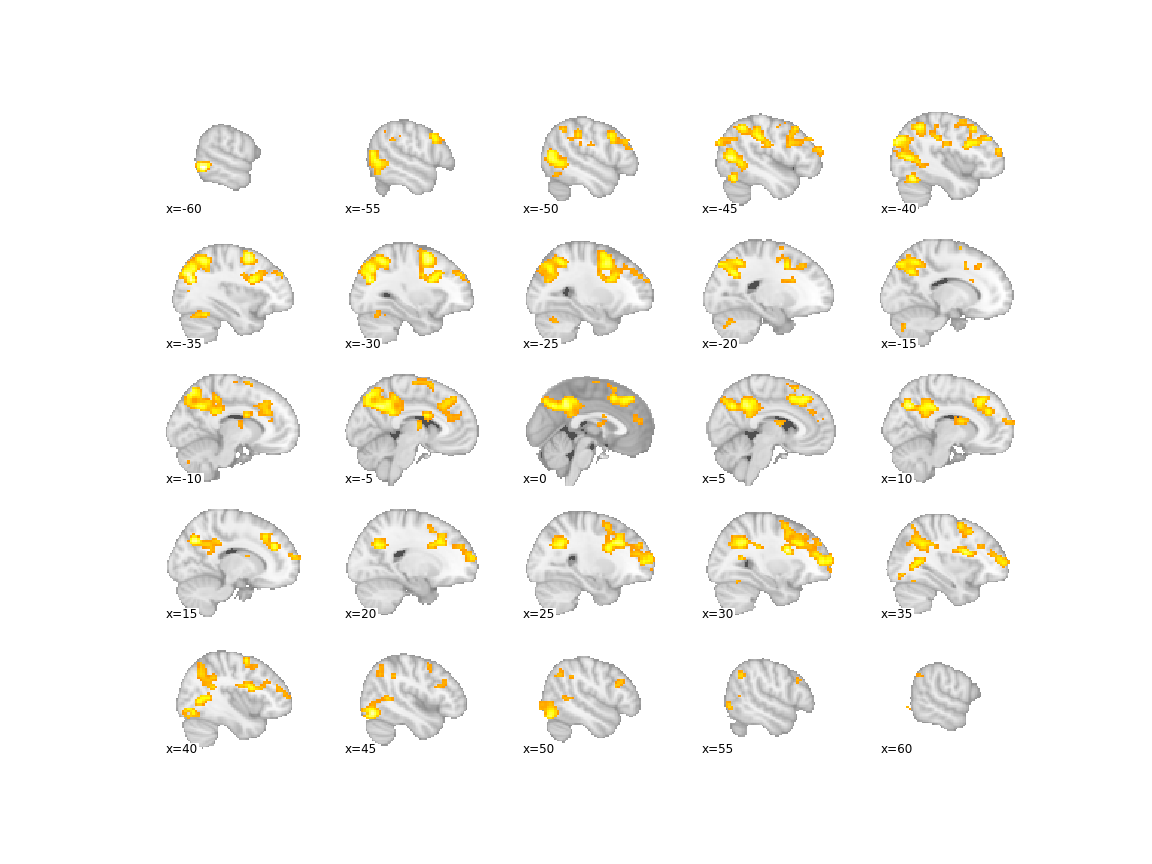
**

**
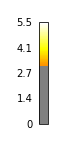
**

**Fig. 5c** *Whole Brain Activation with Top-5 Transitivity (FWE, 3DClustSim;* p = .001, k = 79, corresponding to p<.05, FWE)

*
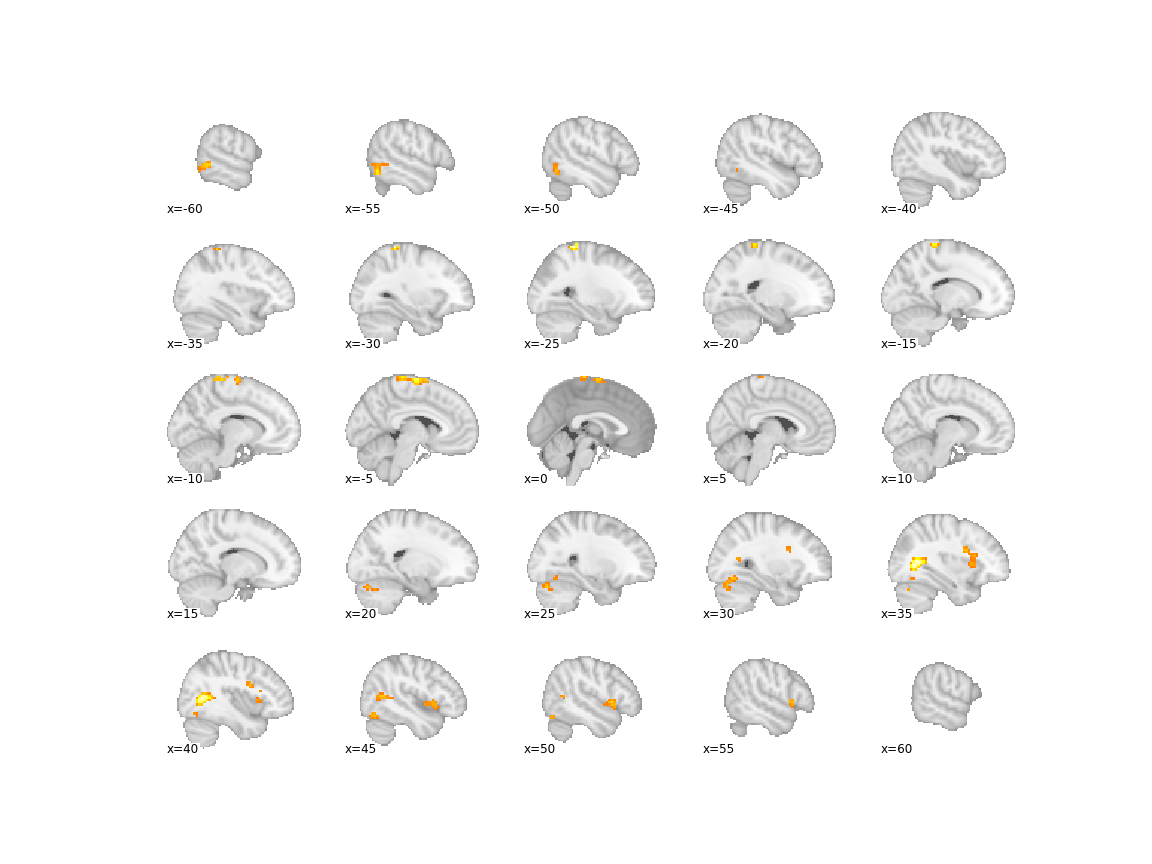
*

**
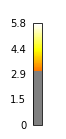
**

**References**

1. Maldjian, J. A., Laurienti, P. J., Kraft, R. A. & Burdette, J. H. An automated method for neuroanatomic and cytoarchitectonic atlas-based interrogation of fMRI data sets. *Neuroimage* **19,** 1233–1239 (2003).

2. Tzourio-Mazoyer, N. *et al.* Automated anatomical labeling of actiavtions in SPM using a macroscopic anatomical parcellation of the MNI MRI single-subject brain. *Neuroimage* **15,** 273–289 (2002).

3. Brett, M., Anton, J., Valabregue, R. & Poline, J. Region of interest analysis using an SPM toolbox. *8th International Conference on Functional Mapping of the Human Brain* **16,** 497 (2002).

4. Falk, E. B. *et al.* Neural Responses to Exclusion Predict Susceptibility to Social Influence. *J. Adolesc. Heal.* **54,** S22-S31 (2014).
